# Anticancer Properties of Novel Rhenium Pentylcarbanato Compounds against MDA-MB-468(HTB-132) Triple Node Negative Human Breast Cancer Cell Lines

**DOI:** 10.9734/BJPR/2014/4697

**Published:** 2015-02-01

**Authors:** Carl Parson, Valerie Smith, Christopher Krauss, Hirendra N. Banerjee, Christopher Reilly, Jeanette A. Krause, James M. Wachira, Dipak Giri, Angela Winstead, Santosh K. Mandal

**Affiliations:** 1 Department of Biology and Pharmaceutical Sciences, Elizabeth City State University, University of North Carolina, Elizabeth City, NC, 27909, USA.; 2 Department of Biomedical Sciences and Pathobiology, Virginia-Maryland Regional College of Veterinary Medicine, Virginia Polytechnic Institute and State University, Blacksburg, Virginia, 24061, USA.; 3 Department of Chemistry, University of Cincinnati, Cincinnati, OH, 45221, USA.; 4 Department of Biology, Morgan State University, Baltimore, MD, 21251, USA.; 5 Department of Chemistry, Morgan State University, Baltimore, MD, 21251, USA.

**Keywords:** Rhenium pentylcarbonato compounds, HTB-132 breast cancer cells, cytotoxicity, trypan blue assay

## Abstract

**Aim:**

To study the efficacy of novel rhenium compounds to treat triple node negative breast cancer.

**Place and Duration:**

Six (6) novel rhenium pentycarbanato compounds (**PC1-6**) were synthesized and triple node negative breast cancer cell lines HTB-132 and Balb/c mouse kidney cell lines were treated with each of them for 48 hours. The results were analyzed by a common trypan blue cell death assay system and statistically analyzed.

**Place and Duration:**

The compounds were synthesized, analyzed and evaluated at the Department of Chemistryof Morgan State University, Baltimore, Maryland and the Pharmaceutical Sciences Department of Elizabeth City State University campus of the University of North Carolina system.

**Methodology:**

The novel rhenium compounds were synthesized from one-pot reactions of Re_2_(CO)_10_ with the corresponding α-diimine ligands in 1-pentanol.The compounds were characterized spectroscopically.

The cell lines were cultured by standard cell culture procedure and treated with each of the six compounds in DMSO for 48 hours with a negative control and a DMSO vehicular control along with a cisplatin positive control.The cytotoxicity was evaluated by standard trypan blue assay and the results were statistically analyzed.

**Results:**

The trypan blueassay reveals that these compounds have significant cytotoxicity against MDA-MB-468 (HTB-132) triple node negative breast cancer cell lines and are less nephrotoxic than cisplatin.

**Conclusion:**

The novel rhenium compounds **PC 1-6** can potentially find applications in the treatment of highly malignant triple node negative breast cancer.

## 1.INTRODUCTION

Breast cancer is the most common cancer among women.There are several drugs developed to treat ER(+) breast cancers but very few or no drugs are there to treat ER(-) especially triple negative breast cancers. Scientists reported success in treating breast cancer with the organic drugs tamoxifen and raloxifene, shrinking advanced tumors with herceptin and treating early cancers with taxol. However, these drugs have severe side effects. For example, tamoxifen causes human endometrial cancer and liver cancer in rats [1,2]. Numerous inorganic anticancer agents have been synthesized [3-5] and only a few of them have been found to be effective against breast cancer. It has been shown that, in combination with other drugs, cisplatin, *cis*-[Pt (NH_3_)_2_Cl_2_], may be effective against breast cancer [6]. Also the cisplatin analogs, oxaliplatin and *DWA*-2114R [7] have been demonstrated to be cytotoxic against breast cancer cells. Cisplatin, carboplatin, oxaliplatin and related metallodrugs are extensively being used in the treatment of a variety of cancers. Unfortunately these drugs are highly toxic and drug-resistance develops over time in cancer cells. These circumstances have led researchers to look for new cytotoxic agents which exhibit less toxicity and no drug resistance. Recently a group of organometallic derivatives of tamoxifen, called ferrocifens have been found to be active against both ER(+) and ER(-) breast cancers [8].

**Fig. 1:**
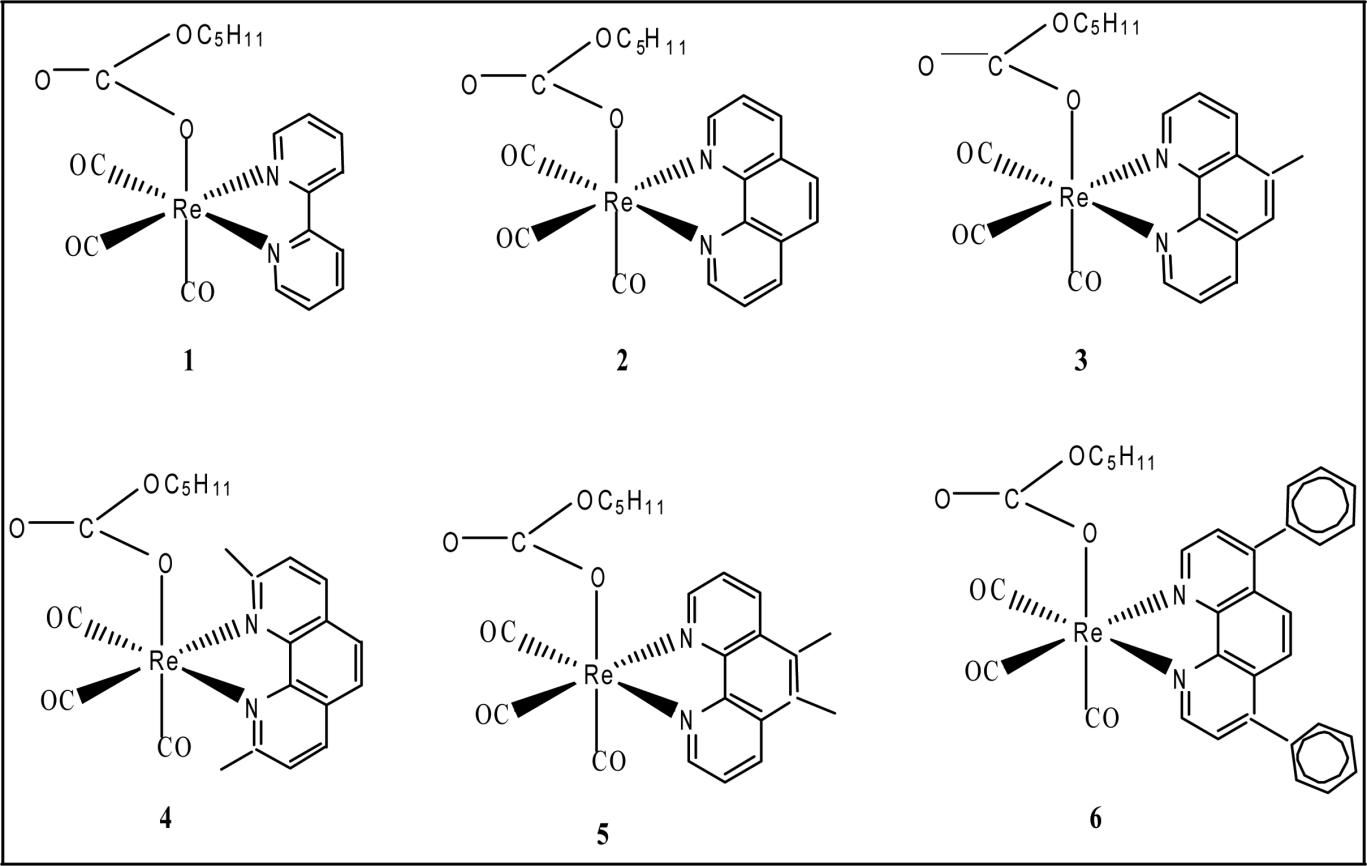
The structural formulae of the PC-series compounds PC 1-6

Several rhenium carbonyl complexes have been reported to be cytotoxic against numerous cancer cell lines [9,10]. Many of these complexes have been found to display cytotoxicity against breast cancer cell lines. However, systematic treatments of these drugs with various breast cancer cell lines including MDA breast cancer cell lines have not been reported.Recently we synthesized several novel rhenium pentylcarbonato compounds [11]. The structural formulae of the compounds (**PC 1-6**) are shown in Fig. 1. Here we report the cytotoxic effects of **PC 1-6** against triple node negative breast cancer cell lines MDA-MB-468 (HTB-132) and Balb/c mouse kidney cell lines.

## 2. MATERIALS AND METHODS

### 2.1 Materials

MDA-MB-468 (HTB-132) triple node negative breast cancer cell lineswere obtained from ATCC (USA), cultured inL-15 medium supplemented with 10% Fetal Bovine Serum(FBS), penicillin and streptomycin antibiotics and grownat 37°C inan incubator. The mouse glomerular mesangialcell line was a kind gift from Dr. C. Reilly of Virginia Tech University, USA. These cells were cultured in RPMI-1640 medium supplemented with 10%FBS, penicillin and streptomycin antibiotics and grown at 37°C in a carbon dioxide incubator.

Synthesis of rhenium pentacarbanato compounds **PC 1-6**: The rhenium compounds **PC 1-6** were synthesized according to published procedure (Mbagu et. al. 2012). In brief, a mixture of Re_2_(CO)_10_ and the corresponding α-diimines in 1:2 mole ratio was refluxed in 1-pentanol while CO_2_ was bubbled through the solutions. The α-diimines in **PC 1–6** are 2,2′-bipyridyl, 1,10-phenanthroline, 5-methyl-1,10-phenanthroline, 2,9-dimethyl-1,10-phenanthroline, 5, 6-dimethyl-1,10-phenanthrolineand4,7-diphenyl-1,10-phenanthroline,respectively. The reaction was discontinued after 24 hours.After cooling to room temperature, the solid pentylcarbonato complexes **PC 1–6** were isolated by filtration.

### 2.2 Cytotoxicity Assay

HTB-132 breast cancer cell lines were grown in six well plates to confluency, the cells were then kept in serum-free medium overnight and then treated with each of drugs**PC1-6** (dissolved in DMSO) along with untreated cells and DMSO vehicular control for 48 hours. Since previous studies with different cancer cell lines [11] showed 50% cell death (IC_50_)dose for these compounds ranged between 2-5 μM, we selected that range as our loading dose. Atrypan blue assay was done to measure cell viability using a standard hemocytometer and light microscope.

### 2.3 Statistical Analysis

Each experiment was repeated three times and statistical t test was used to analyze the results with P<0.05.

## 3. RESULTS

**Fig. 2:**
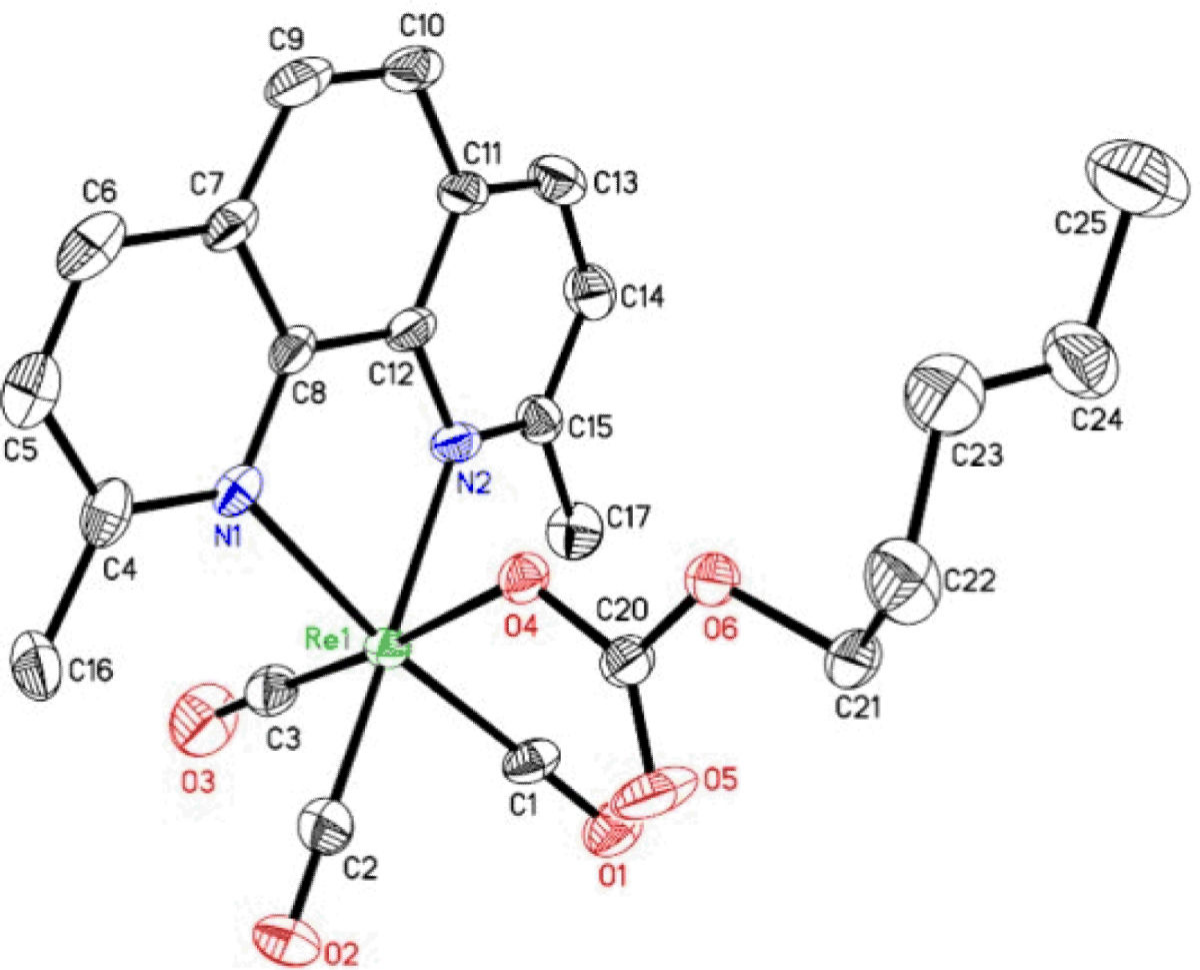
X-ray structure of PC 4

The pentyl carbonato compounds were characterized spectroscopically[11]. Additionally, **PC 1**, **PC 4** and **PC 6** were characterized through X-ray crystal structure determinations. The X-ray structures of **PC 1** and **PC 6** were reported earlier (CCDC 749606 for PC 1 and CCDC 749607 for PC 6 contain the supplementary crystallographic data.These data can be obtained free of charge from The Cambridge Crystallographic Data Centre via www.ccdc.cam.ac.uk/data_request/cif). The X-ray structure of **PC 4** has been carried out recently and is shown in Fig. 2. The crystallographic data and other related information for **PC 4** will be published elsewhere.

Table 1 shows the effects of **PC 1-6** on MDA-MB-468 (HTB-132) and Balb/c mouse kidney cell lines. All the drugs were cytotoxic to the breast cancer cell lines and found to be less nephrotoxic thancisplatin.

## 3. DISCUSSION

Anticancer drugs follow a variety of modes (mechanisms) of action.Cisplatin and related compounds produce intrastrand d(GpG) and d(ApG) adducts [12]. Dinuclear platinum complexes produce DNA (Pt,Pt) interstrand and intrastrand crosslinks [13]. The tetranuclear platinum thiosemicarbazone complex forms DNA interhelical crosslinks [14]. A selective transport mechanism into the cell via transferrin was proposed for ruthenium imidazole complexes [15,16]. The behavior of organometallic metallocenes with DNA is very different to that of cisplatin [17]. Cisplatin and related drugs directly bind (form covalent bond) to the DNA.As a result normal cells specially kidney cells are highly affected resulting in severe toxic side effects.In this study, we found the **PC** compounds to be effective anticancer agents with less nephrotoxicity than cisplatin.

Almost all the **PC**-series compounds (drugs) showed effective cytotoxicity against the triple node negative breast cancer cells for 48 hours exposure.

Triple node negative breast cancer is a fulminant radio-resistant malignant tumor that has almost no known drugs that is an effective chemotherapeutic agent. Further studies are needed with more different node negative breast cancer cell lines and eventually in animal models to come to a conclusion about the real effectiveness of these drugs.Our research demonstrates that the rhenium compounds are effective against triple node negative breast cancer cells. Further studies need to be done regarding the mechanism, nature of cell death and pathway involved in induction of cell death by these compounds.

## Figures and Tables

**Table 1:** The effects of PC 1-6 on MDA-MB-468 (HTB-132) and Balb/c mouse kidney cell lines

Drugs	Breast Cancer HTB-132 cell lines	Mouse Mesangial cell lines
RPC1	3±2.5	5±2.5
RPC2	2±1.5	5±3.5
RPC3	3±2.5	4±2.5
RPC4	2±2.5	5±5.5
RPC5	3±4.5	4±2.5
RPC6	4±6.5	5±1.5
Cisplatin	5±2.5	1±2.5

IC- 50 (mean ± S.D.) refers to the concentration required to have 50% cell-growth inhibition; DMSO was inactive in both the cell lines tested.
